# Cang-Ai Volatile Oil Ameliorates Chronic Unpredictable Mild Stress-Induced Depression-like Symptoms in Rats by Regulating NT/Trk Signaling Pathway

**DOI:** 10.3390/ph19050751

**Published:** 2026-05-11

**Authors:** Mingqin Shi, Haimei Zhou, Xiangdian Xiao, Chengting Jiang, Lei Pan, Xiaoman Lv, Tengfei Qian, Dongdong Qin

**Affiliations:** 1 Key Laboratory of Traditional Chinese Medicine for Prevention and Treatment of Neuropsychiatric Diseases, Yunnan University of Chinese Medicine, Kunming 650500, China; shimingqin1998@163.com (M.S.); 17773572364@163.com (H.Z.); jiangchengting@ynucm.edu.cn (C.J.); lxm.cc@foxmail.com (X.L.); 2 First Clinical Medical College, Yunnan University of Chinese Medicine, Kunming 650500, China; 3 United Graduate School, China Academy of Chinese Medical Sciences, Suzhou 215000, China; 4 School of Traditional Chinese Medicine, Qujing University of Medicine & Health Sciences, Qujing 655100, China; 15911428866@163.com; 5 Second Clinical Medical College, Yunnan University of Chinese Medicine, Kunming 650500, China; fanxieye@163.com; 6 College of Pharmaceutical Science, Dali University, Dali 671000, China; 7 The People’s Hospital of Mengzi, The Affiliated Hospital of Yunnan University of Chinese Medicine, Mengzi 661100, China

**Keywords:** Cang-ai volatile oil, depression, chronic unpredictable mild stress, NT/Trk signaling pathway, potential mechanism

## Abstract

**Background:** Cang-ai volatile oil (CAVO) is a traditional Chinese medicine with properties that soothe the liver and alleviate depression. CAVO is widely utilized in the field of antidepressant research and has surfaced as a possible treatment for depression. Depression is a common affective disorder and effective treatment methods are still limited. CAVO is effective in treating depression; however, the exact mechanism is still unclear. This study aimed to explore the likely mechanism by which CAVO reduces symptoms of depression in rats exposed to chronic unpredictable mild stress (CUMS). **Methods:** We established a CUMS model in Sprague–Dawley rats and administered CAVO via nebulization to evaluate its therapeutic effect. Behavioral and histology tests were conducted to evaluate brain tissue damage. We utilized metabolomics combined with proteomics to analyze the effects of CAVO. We then assessed molecular validation to further clarify the molecular mechanism of its activity. **Results:** In CUMS model rats, inhaling aerosolized CAVO reduced brain pathology and depression-like behaviors. CAVO changed serum levels of inflammatory cytokines and neurotrophic factors. Biomarkers linked to CAVO’s antidepressant effects were found via metabolomics. Functional analyses highlighted key molecular players such as TrkB, and CREB, and a close association with the antidepressant action of CAVO was confirmed. **Conclusions:** This study reveals that CAVO reduces depression-like behaviors in CUMS rats by regulating the NT/Trk signaling pathway. These results demonstrate CAVO’s therapeutic potential and lay the groundwork for future studies and the creation of depressive treatments.

## 1. Introduction

Depression is a prevalent emotional disease distinguished by a sustained and significantly diminished mood that is incongruent with an individual’s circumstances, accompanied by loss of interest and reduced activity levels [1,2]. Patients commonly present with symptoms such as weight changes, psychomotor retardation, decreased physical activity, mental fatigue, sleep disturbances, low self-esteem, impaired concentration, and recurrent suicidal ideation [3,4]. Depression is associated with high prevalence, high recurrence, substantial disability, and elevated mortality. The World Health Organization reports that roughly 322 million individuals worldwide are affected by depression [5]. Major depressive illness is predicted to rank as the second most common cause of disability globally by 2030 [6]. Therefore, greater emphasis on the treatment of depression is warranted.

Ketamine, a non-competitive N-methyl-D-aspartate receptor (NMDAR) antagonist, has demonstrated rapid onset and sustained (up to several days) antidepressant effects in patients with depression who are refractory to traditional antidepressant medications [7]. Its antidepressant effect has been recognized by multiple international experts [8]. But some drawbacks of commonly used antidepressants, such as a high frequency of side effects, a delayed therapeutic beginning, and an elevated risk of suicidal thoughts in certain patients, restrict their clinical utility [9,10]. High medical expenses also place a significant financial strain on families and society as a whole. Consequently, there is an urgent need to identify treatments that are simple, safe, and effective.

Cang-ai volatile oil (CAVO) is a representative compound volatile oil formulation in traditional Chinese aromatherapy, developed from the extensive clinical experience of Professor Xiong Lei, a renowned traditional Chinese medicine physician from Yunnan Province. CAVO is composed of aromatic herbs such as Atractylodes, Artemisia, Agastache, Phellodendron, and clove, which are traditionally described as possessing pungent, dispersing, and penetrating properties. Its active constituents are extracted through a standardized process to form the final formulation. Clinically, children’s upper respiratory tract infections and mental disturbances are frequently treated with CAVO, including anxiety disorders, menopausal depression, and postpartum depression, with reported favorable therapeutic outcomes [11,12]. As an aromatic essential oil, CAVO can readily penetrate the blood–brain barrier, allowing direct access to the brain via the nasal cavity and potentially enabling rapid central nervous system effects. Its distinctive aroma may induce a sense of pleasure, and according to traditional Chinese medicine theory, it is believed to regulate qi and alleviate depressive symptoms. Moreover, compound herbal essential oils exhibit multi-target pharmacological activities and a broad spectrum of biological effects.

Compared with conventional Western antidepressants that typically target a single pathway, CAVO is associated with fewer adverse reactions [13]. Taken together, these characteristics indicate that CAVO has a lot of promise for treating and preventing depression and merits more research. This study aimed to investigate the potential mechanism by which CAVO mitigates depressive symptoms in rats subjected to CUMS.

## 2. Results

### 2.1. Body Weight Determination

**Figure 1:**
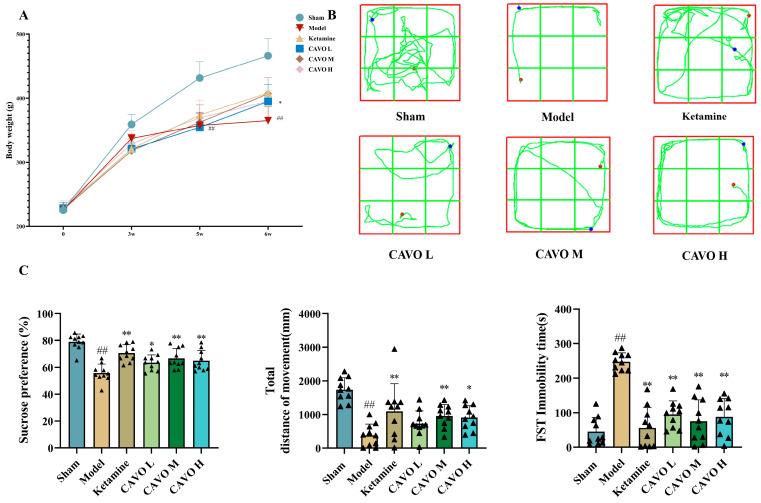
Effects of CAVO on weight change and behavioral outcomes. (**A**) Weight changes during the experimental period. (**B**) The movement trajectories of rats. Red dot: The initial position of the rats at the beginning of the experiment. Blue dot: The final position of the rats at the end of the experiment. (**C**) Comparison of behavioral outcomes among different groups after treatment. There were 10 animals in each group. Data were presented as individual data points with mean ± SD. Compared with the sham group, ^##^ *p* < 0.01. Compared with the model group, * *p* < 0.05; ** *p* < 0.01. The data of sucrose preference and total distance of movement were normally distributed and had homogeneous variances. Therefore, one-way ANOVA was used. Sucrose preference: F(5, 54) = 13.70, *p* = 1.23 × 10^−8^; total distance of movement: F(5, 54) = 9.41, *p* = 1.72 × 10^−6^. Post hoc pairwise comparisons were performed using the LSD method. Sucrose preference: compared with the sham group, *p*_Model_ = 1.67 × 10^−10^; compared with the model group, *p*_Ketamine_ = 6.41 × 10^−6^, *p*_CAVO L_ = 1.19 × 10^−2^, *p*_CAVO M_ = 5.58 × 10^−4^, *p*_CAVO H_ = 3.03 × 10^−3^. Total distance of movement: compared with the sham group, *p*_Model_ = 2.39 × 10^−8^; compared with the model group, *p*_Ketamine_ = 1.16 × 10^−3^, *p*_CAVO L_ = 1.02 × 10^−1^, *p*_CAVO M_ = 7.59 × 10^−3^, *p*_CAVO H_ = 1.43 × 10^−2^. The variance of FST was uneven, and the Welch test was used. FST: F(5, 24.69) = 51.67, *p* = 2.78 × 10^−12^. Post hoc pairwise comparisons were performed using Tamhane’s T2. Compared with the sham group, *p*_Model_ = 6.48 × 10^−13^; compared with the model group, *p*_Ketamine_ = 3.93 × 10^−12^, *p*_CAVO L_ = 3.33 × 10^−9^, *p*_CAVO M_ = 1.07 × 10^−10^, *p*_CAVO H_ = 9.44 × 10^−10^.

To evaluate the overall physiological effects of CAVO on CUMS-induced depression, body weight was monitored at 0, 3, 5, and 6 weeks post-modeling. Intergroup comparison results are shown in Figure 1A. Throughout the experiment, the rats in the sham group’s body weight, who were fed a regular diet, significantly increased. Five weeks after modeling, the body weight of the rats in the model group was significantly lower than that of the sham group (*p* < 0.01). The positive pharmaceutical ketamine group’s body weight considerably increased after seven days of continuous dose when compared to the model group (*p* < 0.01), while the CAVO M group, CAVO H group, and CAVO L group also showed increases in body weight (*p* < 0.05).

### 2.2. CAVO Improves CUMS-Induced Depression-like Behavior in Rats

To assess the antidepressant-like effects of CAVO on CUMS-induced depressive behaviors, we performed the sucrose preference test (SPT), open-field test (OFT), and forced swim test (FST). Rats’ movement trajectories during the open-field test are displayed in Figure 1B. Rats in the sham group traveled more around the center and showed a strong desire to explore. The rats in the CUMS group, on the other hand, only walked back and forth in the periphery region and moved considerably less in the middle area, suggesting that they were anxious. The treatment of ketamine and CAVO dramatically decreased the CUMS rats’ anxiety and stereotyped behaviors.

The behavioral results after drug intervention are shown in Figure 1C. Relative to the model group, the depressive behaviors in the ketamine group and the CAVO treatment group exhibited improvement, suggesting that depressive-like behavior in rats ameliorated following CAVO therapy.

### 2.3. CAVO Can Improve CUMS-Induced Hippocampal Tissue Damage

**Figure 2:**
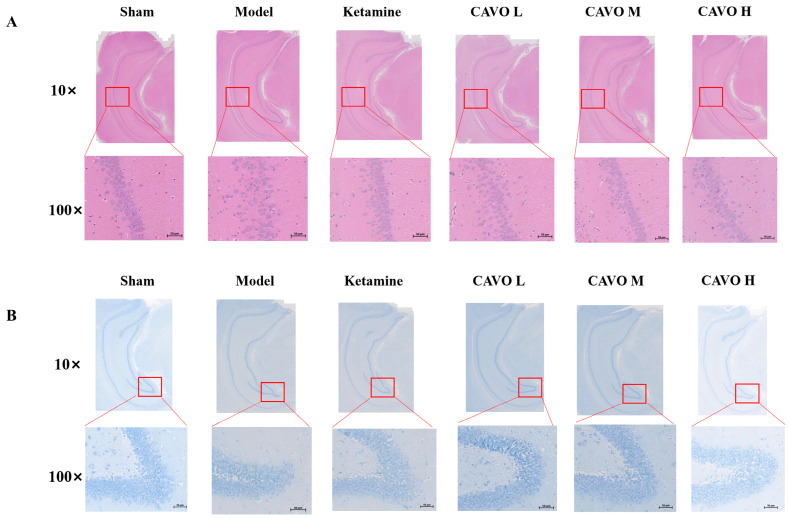
Histopathology changes in the hippocampus were investigated by histopathological examination (100×). (**A**) HE staining of the hippocampal tissue. (**B**) Nissl staining of the hippocampal tissue. The red box shows extensive atrophy and deformation of hippocampal neurons. (*n* = 3).

To determine whether CAVO treatment ameliorates CUMS-induced neuronal damage, hematoxylin and eosin (HE) staining and Nissl staining were performed on hippocampal sections. Figure 2A illustrates the HE staining of the hippocampus. The CA1 had a large number of neurons in the sham group. The cell morphology remained intact, and the hippocampal neurons were systematically and firmly organized. The CUMS group, on the other hand, had fewer hippocampus neurons and poorly organized, loosely packed cells. Neuronal cells had abnormal morphology and were reduced and distorted. Overall, the CUMS group showed significant neuronal injury. Compared to the CUMS group, the ketamine group had a greater number of hippocampal neuronal cells. The hippocampal neurons had homogeneous cytoplasmic staining, clean cell membranes, and a tidy arrangement. The findings indicate that in the ketamine group, the neuronal damage was partially repaired. Additionally, CAVO aromatherapy helped to heal damaged neurons. The CAVO groups showed more neuronal cells in the CA1 areas than the CUMS group. Nuclear pyknosis was still present in a small percentage of cells, but overall, things were much better than in the CUMS group. Figure 2B displays the results of the Nissl staining. Numerous Nissl structures were visible in the cytoplasm of the sham group, and the hippocampal neurons were densely packed. The hippocampus neurons in the CUMS group, on the other hand, were loosely organized, had fewer Nissl bodies, and some wrinkled cells. These findings imply that protein production was impaired and hippocampus neurons were destroyed in the CUMS group. After ketamine and CAVO treatment, the neurons were placed closer together than in the CUMS group. Following ketamine and CAVO treatment, many Nissl bodies were found in the cytoplasm.

### 2.4. Metabolomics Results of CAVO Intervention on CUMS Rats

**Figure 3:**
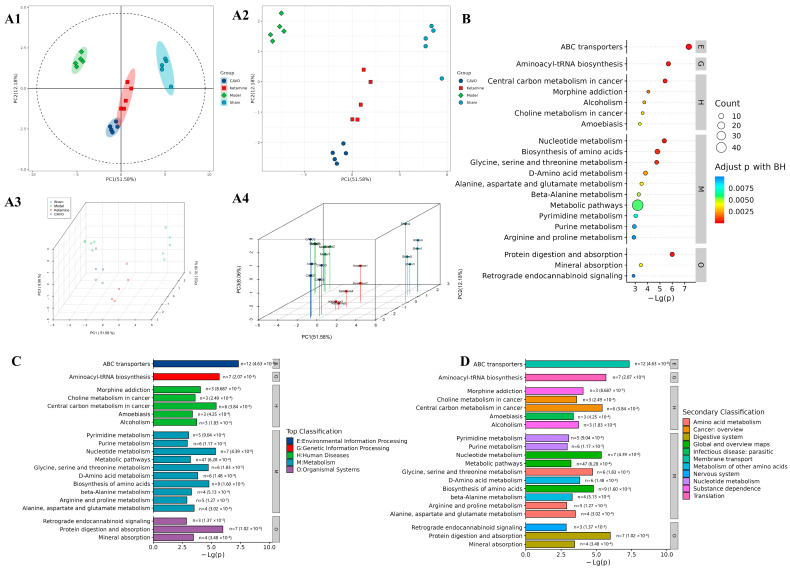
Metabolomic analysis of rat hippocampal tissue. (**A**) Multivariate statistical analysis of metabolomics data. (**A1**) PCA score plot; (**A2**) PCA load plot; (**A3**) PLS-DA score plot; (**A4**) gravel plot. (**B**) Metabolome KEGG pathway bubble chart (top 20). (**C**) KEGG pathway enrichment bar chart (top 20). (**D**) KEGG pathway enrichment bar chart (top 20). Level 1 pathway classification: metabolism (M); genetic information processing (G). Environmental information processing (E); organismal systems (O); human diseases (H) (*n* = 5, the x-axis represents the pathway impact value calculated by topological analysis. A higher impact value indicates a more important metabolic pathway).

To identify metabolic alterations associated with CAVO treatment and uncover potential biomarkers underlying its antidepressant effects, untargeted metabolomics analysis was performed on hippocampal tissues. Metabolite ion peaks were retrieved utilizing MSDIAL5 software, resulting in a total of 39,950 ion peaks gathered. The PCA clustering analysis results after processing are shown in Figure 3A. A distinct separation trend between the sham group, model group, ketamine group, and CAVO group can be seen in the PCA scoring table. Additionally, the ketamine group and CAVO group were closer to the sham group compared to the model group, indicating that the administration improved the metabolic function of CUMS rats and promoted their recovery to a normal healthy state.

For biomarker analysis, using models such as PCA and OPLS-DA, compounds with VIP > 1 were screened for results with *p* < 0.05 corrected using the FDR method to identify 25 differentially expressed metabolites associated with depression, including 8 upregulated and 17 downregulated metabolites (Table 1). Online databases were used to identify metabolites that were differentially expressed. The results indicated that the therapeutic effect of CAVO is due to its regulation of these metabolites. The model group’s levels of eight metabolites were noticeably greater than those of the sham group: 1-(diethylamino) ethanol, indane, cycluron, N-stearoyl taurine, 4-ethynylaniline, 3-oxohexadecanoic acid, 3H-indole-3-propanoic acid, α-amino-(3H-indole-3-propanoic acid), α-amino-, and (9Z)-2-hydroxy-2-methyl-9-octadecenoic acid (CHEBI:79180). Seventeen metabolites showed significantly reduced levels: N-acetylneuraminic acid, choline, (Z)-eicosapentaenoic acid ester (PE [P-18:0/18:1]), calicoferol G, glutathione, gallic acid, UDP-glucose, oboflavanone B, persicaxanthin, sedoheptulose 7-phosphate, phenyl 2-acetamido-2-deoxy-α-D-glucopyranoside, graveolide, aspartate, reduced glutathione, L-histidine, L-arginine, phosphatidylcholine 12-hydroxy-9Z-hexadecenoic acid (PC [18:2/18:2]).

The differentially expressed metabolites found in the tissue were subjected to pathway enrichment analysis to understand their roles in metabolic pathways. The results are shown in Figure 3B–D. KEGG enrichment was performed at the Level 1 classification (top 20), with the following significant metabolic pathways identified: alanine, aspartate, and glutamate metabolism; ATP-binding cassette transporters; aminoacyl-tRNA biosynthesis; nucleotide metabolism; and amino acid biosynthesis (*p* < 0.01).

### 2.5. Proteomics Results of CAVO Intervention on CUMS Rats

**Figure 4:**
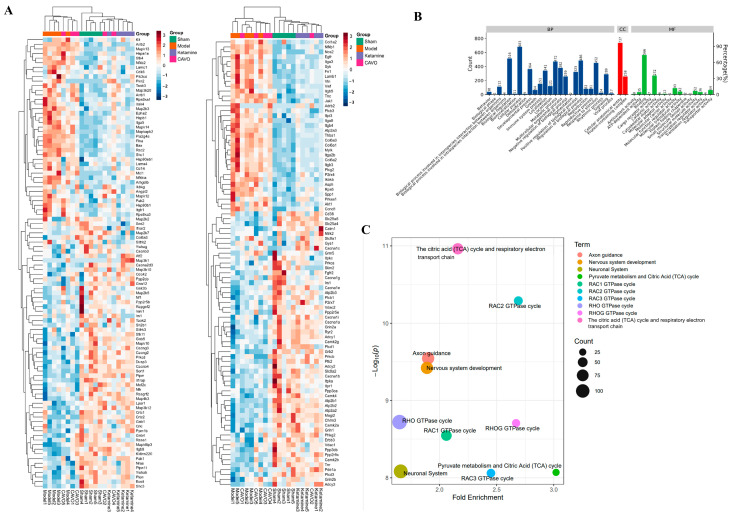
Proteomics analysis of rat hippocampal tissue. (**A**) Heat map of differentially expressed proteins in the sham, model, ketamine, and CAVO groups. (**B**) GO analysis of differentially expressed proteins in CAVO callback. Note: biological process (BP), molecular function (MF), and cellular component (CC) are represented by different colors. Each column is labeled with the specific count. (**C**) REACTOME pathway enrichment bubble chart (top 10) of proteins with significant differences between the sham, model, ketamine, and CAVO groups. This allows the three parameters—*p*-value, fold enrichment, and count—to be displayed simultaneously in a single figure. (*n* = 5, the x-axis represents fold enrichment, and the y-axis represents the negative logarithmic transformation of the *p*-value. The size of the circles represents the count, and different colors indicate different pathways).

To investigate the protein-level changes associated with CAVO treatment and elucidate the molecular mechanisms underlying its antidepressant effects, proteomics analysis was conducted on hippocampal tissues. In the examination of notable disparities in protein expression, the default test used for multiple group comparisons was a one-way ANOVA. Proteins with *p* < 0.05 were selected as differentially expressed proteins, and 3261 significantly different proteins were detected. Among these, 200 differentially expressed proteins related to depression were screened. As shown in Figure 4A, in the CUMS group, 124 proteins were upregulated, and 76 proteins were downregulated. In complete clustering, red hues signify elevated relative expression levels, whereas blue hues denote diminished relative expression levels.

The functional distribution of CAVO depression differentially expressed proteins was investigated using GO annotation analysis. The differentially expressed proteins for CAVO recall are depicted in the GO enrichment pathway diagram in Figure 4B. Compared with the model group, the CAVO group mainly participated in biological processes such as defense reactions and immune system processes; cellular components mainly included neural cell connections; and molecular functions mainly included anti-inflammatory and antioxidant activities.

The hypergeometric distribution test technique was used to conduct functional enrichment analysis of differentially expressed proteins in the comparison group. *p*-values are used to express the results; substantial functional enrichment is indicated by values less than 0.05, and more significant functional enrichment is shown by lower values. Figure 4C shows the top 10 pathways with significant enrichment, presented as a bubble chart.

### 2.6. Combined Metabolic and Proteomic Analysis Results

**Figure 5:**
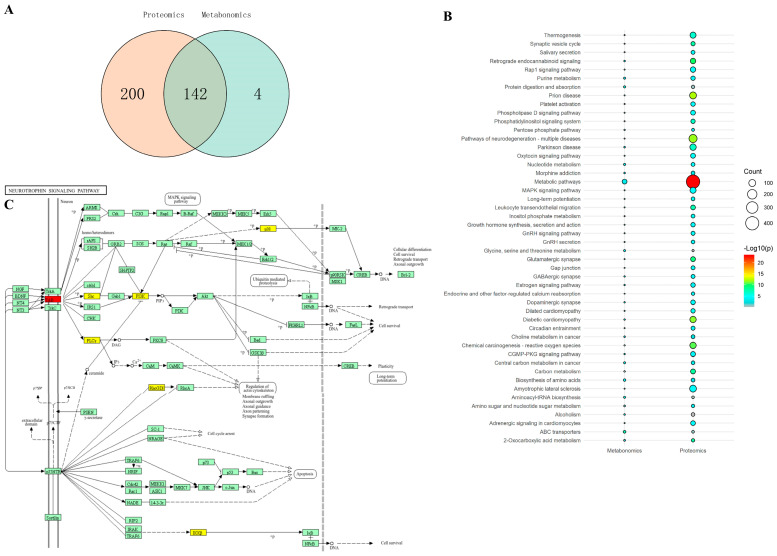
Combined metabolic and proteomic analysis. (**A**) Venn diagram of pathways in proteomics and metabolomics. (**B**) Enriched pathways in proteomics and metabolomics. (**C**) Protein pathway annotation diagram of the neurotrophic factor pathway.

To integrate the metabolomics and proteomics findings and identify converging pathways potentially responsible for the antidepressant effects of CAVO, joint pathway analysis was performed. Figure 5A shows a Venn diagram of pathways in proteomics and metabolomics. Figure 5B shows the enriched pathways. There are 342 pathways jointly regulated by proteomics and metabolomics in depression, and CAVO can modulate 142 of them, including the MAPK pathway, interactions between neuroactive ligands and receptors, glutamatergic synapses, protein digestion and absorption, and ABC transporter signaling pathways. CAVO can regulate these pathways at the protein and metabolic levels to exert its antidepressant effects.

Figure 5C shows the annotation of the pathway, where the boxes represent proteins/genes and the dots represent metabolites. Red-filled boxes indicate proteins with upregulation, yellow-filled boxes indicate proteins with downregulation, and green boxes indicate background proteins, as shown in Figure 5C. Following CAVO intervention therapy in CUMS model rats, TrkB protein expression was significantly upregulated in the neurotrophic factor signaling pathway, while p38, Shc, PI3K, PLC-γ, RhoGDI, and IKKβ protein expression was downregulated.

### 2.7. ELISA Results of CAVO on CUMS Rat Serum

**Figure 6:**
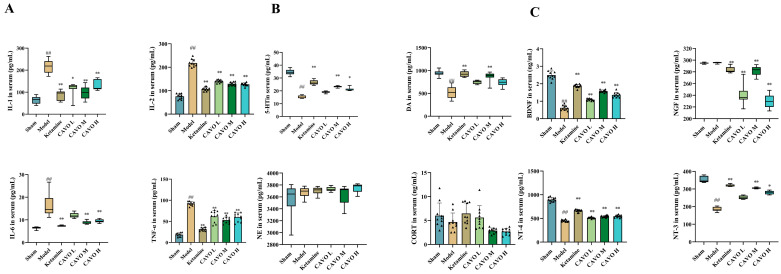
The contents of stress hormone corticosterone (CORT), inflammatory factors (IL-1, IL-2, IL-6, TNF-α), neurotransmitters (5-HT, DA, NE), and neurotrophic factors (NGF, BDNF, NT-3, NT-4) in serum. (**A**) The content of IL-1, IL-2, IL-6, TNF-α in serum. (**B**) The content of 5-HT, DA, NE, CORT in serum. (**C**) The content of NGF, BDNF, NT-3, NT-4 in serum. Each group had 10 animals. Data that conformed to a normal distribution were presented as mean ± SD. Data that did not follow a normal distribution were expressed as median and IQR. Compared with the sham group, ^##^ *p* < 0.01. Compared with the model group, * *p* < 0.05; ** *p* < 0.01. The data of IL-2, TNF-α, CORT, BDNF, and NT-4 followed a normal distribution. The variance in the data was uneven, and Welch tests were used. IL-2: F(5, 24.96) = 113.76, *p* = 2.39 × 10^−16^; TNF-α: F(5, 24.72) = 270.54, *p* = 9.64 × 10^−21^; CORT: F(5, 23.87) = 10.42, *p* = 2.15 × 10^−5^, BDNF: F(5, 24.61) = 185.34, *p* = 1.07 × 10^−18^, NT-4: F(5, 24.88) = 180.95, *p* = 1.01 × 10^−18^. Post hoc pairwise comparisons were performed using Tamhane’s T2. IL-2: compared with the sham group, *p*_Model_ = 1.07 × 10^−12^; compared with the model group, *p*_Ketamine_ = 5.67 × 10^−10^, *p*_CAVO L_ = 1.28 × 10^−7^, *p*_CAVO M_ = 1.34 × 10^−8^, *p*_CAVO H_ = 1.56 × 10^−8^. TNF-α: compared with the sham group, *p*_Model_ = 0.00 × 10^0^; compared with the model group, *p*_Ketamine_ = 3.33 × 10^−15^, *p*_CAVO L_ = 4.32 × 10^−4^, *p*_CAVO M_ = 3.54 × 10^−9^, *p*_CAVO H_ = 8.85 × 10^−6^. CORT: compared with the sham group, *p*_Model_ = 9.71 × 10^−1^; compared with the model group, *p*_Ketamine_ = 6.49 × 10^−1^, *p*_CAVO L_ = 9.99 × 10^−1^, *p*_CAVO M_ = 2.09 × 10^−1^, *p*_CAVO H_ = 1.35 × 10^−1^. BDNF: compared with the sham group, *p*_Model_ = 2.87 × 10^−11^; compared with the model group, *p*_Ketamine_ = 1.65 × 10^−12^, *p*_CAVO L_ = 7.12 × 10^−6^, *p*_CAVO M_ = 9.08 × 10^−10^, *p*_CAVO H_ = 1.73 × 10^−8^. NT-4: compared with the sham group, *p*_Model_ = 2.40 × 10^−11^; compared with the model group, *p*_Ketamine_ = 7.24 × 10^−12^, *p*_CAVO L_ = 1.37 × 10^−5^, *p*_CAVO M_ = 1.93 × 10^−7^, *p*_CAVO H_ = 2.62 × 10^−6^. The data of IL-1, IL-6, 5-HT, DA, NE, NGF, and NT-3 were not normally distributed, and Kruskal–Wallis tests with post hoc analysis were used. IL-1: H = 36.51, *p* = 7.53 × 10^−7^; IL-6: H = 55.44, *p* < 0.01; 5-HT: H = 57.38, *p* = 4.22 × 10^−11^; DA: H = 44.38, *p* = 1.94 × 10^−8^; NE: H = 9.71, *p* = 8.39 × 10^−2^; NGF: H = 52.60, *p* = 4.07 × 10^−10^; NT-3: H = 57.35, *p* = 4.29 × 10^−11^. Post hoc test results of IL-1: compared with the sham group, *p*_Model_ = 1.88 × 10^−8^; compared with the model group, *p*_Ketamine_ = 1.89 × 10^−5^, *p*_CAVO L_ = 2.12 × 10^−2^, *p*_CAVO M_ = 4.61 × 10^−4^, *p*_CAVO H_ = 4.61 × 10^−4^. Post hoc test results of IL-6: compared with the sham group, *p*_Model_ < 0.01; compared with the model group, *p*_Ketamine_ < 0.01, *p*_CAVO L_ = 3.37 × 10^−1^, *p*_CAVO M_ < 0.01, *p*_CAVO H_ = 8.00 × 10^−3^. Post hoc test results 5-HT: compared with the sham group, *p*_Model_ = 1.53 × 10^−10^; compared with the model group, *p*_Ketamine_ = 3.03 × 10^−7^, *p*_CAVO L_ = 2.00 × 10^−1^, *p*_CAVO M_ = 1.22 × 10^−4^, *p*_CAVO H_ = 1.04 × 10^−2^. Post hoc test results of DA: compared with the sham group, *p*_Model_ = 9.33 × 10^−8^; compared with the model group, *p*_Ketamine_ = 5.73 × 10^−7^, *p*_CAVO L_ = 6.34 × 10^−2^, *p*_CAVO M_ = 4.80 × 10^−5^, *p*_CAVO H_ = 6.16 × 10^−2^. Post hoc test results of NE: compared with the sham group, *p*_Model_ = 6.54 × 10^−1^; compared with the model group, *p*_Ketamine_ = 6.77 × 10^−1^, *p*_CAVO L_ = 2.39 × 10^−1^, *p*_CAVO M_ = 1.00 × 10^0^, *p*_CAVO H_ = 2.54 × 10^−2^. Post hoc test results of NGF: compared with the sham group, *p*_Model_ = 6.31 × 10^−1^; compared with the model group, *p*_Ketamine_ = 6.50 × 10^−3^, *p*_CAVO L_ = 4.08 × 10^−7^, *p*_CAVO M_ = 3.65 × 10^−3^, *p*_CAVO H_ = 1.96 × 10^−8^. Post hoc test results of NT-3: compared with the sham group, *p*_Model_ = 1.53 × 10^−10^; compared with the model group, *p*_Ketamine_ = 3.03 × 10^−7^, *p*_CAVO L_ = 1.98 × 10^−1^, *p*_CAVO M_ = 1.22 × 10^−4^, *p*_CAVO H_ = 1.06 × 10^−2^.

To assess the effects of CAVO on peripheral inflammatory cytokines and neurotrophic factors, ELISA was carried out on serum samples. Serum levels of TNF-α, IL-1β, IL-2, and IL-6 were significantly higher in the model group than in the sham group, as seen in Figure 6A (*p* < 0.01). Treatment with ketamine, CAVO L, CAVO M, and CAVO H reduced the serum concentrations of these inflammatory cytokines relative to the model group, with the most pronounced decrease observed in the ketamine group. In the CAVO treatment group, the CAVO M group showed the most significant decrease.

Figure 6B presents the serum levels of neurotransmitters. Compared to the sham group, the model group’s 5-HT and DA concentrations were noticeably lower (*p* < 0.01). In contrast, all treatment groups (ketamine, CAVO L, CAVO M, CAVO H) demonstrated significantly higher serum levels of 5-HT compared to the model group. There were no discernible statistically significant variations in serum NE and CORT levels between the model group and the sham group (*p* > 0.05), nor between any treatment group and the model group (*p* > 0.05).

The serum concentrations of BDNF, NT-3, and NT-4 are illustrated in Figure 6C. The model group’s levels of these neurotrophic factors were noticeably lower than those of the sham group (*p* < 0.01). Serum levels of BDNF, NT-3, and NT-4 were noticeably higher in all treatment groups than in the model group (*p* < 0.01), with the CAVO M group again showing the most marked increase. Serum NGF levels did not differ significantly between the model and sham groups (*p* > 0.05). However, the ketamine group displayed lower serum NGF levels than the model group (*p* < 0.01), while the CAVO L, CAVO M, and CAVO H groups demonstrated significantly reduced NGF levels relative to the model group (*p* < 0.01).

### 2.8. RT-PCR Results of CAVO on the Prefrontal Cortex of CUMS Rats

**Figure 7:**
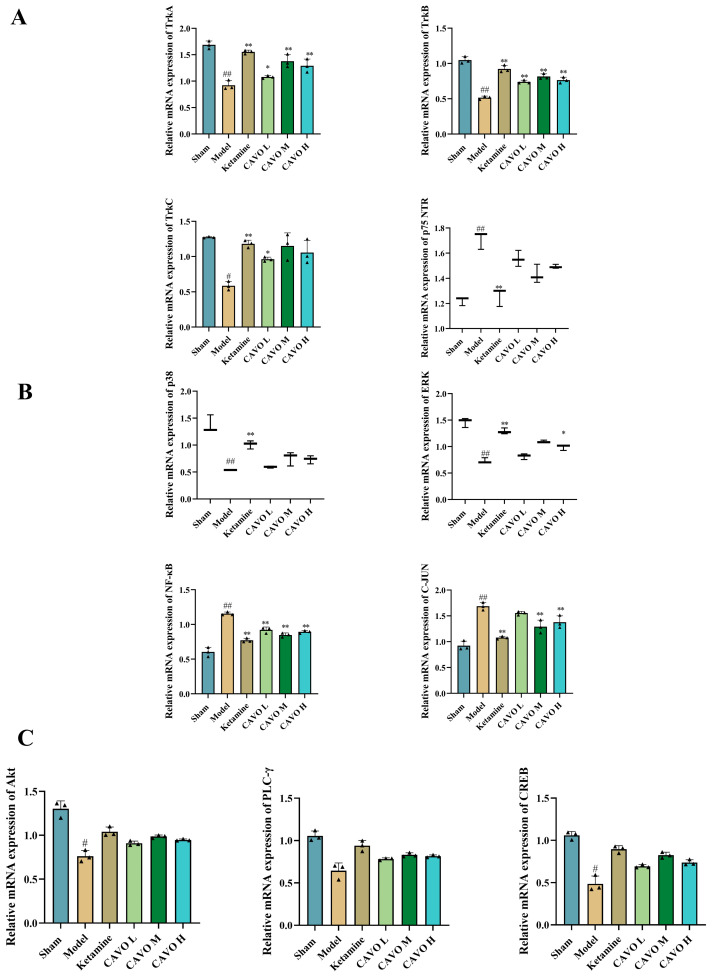
RT-PCR results of CAVO on the prefrontal cortex of CUMS rats (**A**) The mRNA expression levels of TrkA, TrkB, TrkC, and P75NTR in the prefrontal cortex of CUMS rats. (**B**) The expression levels of p38, ERK1/2, NF-κB, and c-Jun mRNA in the prefrontal cortex of CUMS rats. (**C**) The expression levels of Akt, PLC-γ, and CREB mRNA in the prefrontal cortex of CUMS rats. Each group had 3 animals. Data that conformed to a normal distribution were presented as mean ± SD. Data that did not follow a normal distribution were expressed as median and IQR. Compared with the sham group, ^#^ *p* < 0.05, ^##^ *p* < 0.01. Compared with the model group, * *p* < 0.05; ** *p* < 0.01. The data of TrkA, TrkB, TrkC, NF-κB, c-Jun, Akt, PLC-γ and CREB followed a normal distribution. The data of TrkA, TrkB, NF-κB and c-Jun had homogeneous variances. Therefore, one-way ANOVA was used. TrkA: F(5, 12) = 32.15, *p* = 1.50 × 10^−6^; TrkB: F(5, 12) = 64.80, *p* = 2.90 × 10^−8^; NF-κB: F(5, 12) = 67.25, *p* = 2.35 × 10^−8^; c-Jun: F(5, 12) = 32.15, *p* = 1.50 × 10^−6^. Post hoc pairwise comparisons were performed using the LSD method. TrkA: compared with the sham group, *p*_Model_ = 1.71 × 10^−7^; compared with the model group, *p*_Ketamine_ = 1.33 × 10^−6^, *p*_CAVO L_ = 4.95 × 10^−2^, *p*_CAVO M_ = 3.51 × 10^−5^, *p*_CAVO H_ = 2.34 × 10^−4^. TrkB: compared with the sham group, *P*_Model_ = 1.05 × 10^−9^; compared with the model group, *P* _Ketamine_ = 2.24 × 10^−8^, *p*_CAVO L_ = 1.27 × 10^−5^, *p*_CAVO M_ = 6.32 × 10^−7^, *p*_CAVO H_ = 4.49 × 10^−6^. NF-κB: compared with the sham group, *p*_Model_ = 6.27 × 10^−10^; compared with the model group, *p*_Ketamine_ = 3.93 × 10^−8^, *p*_CAVO L_ = 7.78 × 10^−6^, *p*_CAVO M_ = 4.60 × 10^−7^, *p*_CAVO H_ = 2.53 × 10^−6^. C-Jun: compared with the sham group, *p*_Model_ = 1.71 × 10^−7^; compared with the model group, *p*_Ketamine_ = 1.96 × 10^−6^, *p*_CAVO L_ = 8.72 × 10^−2^, *p*_CAVO M_ = 1.29 × 10^−4^, *p*_CAVO H_ = 9.84 × 10^−4^. The data of TrkC, Akt, PLC-γ and CREB had uneven variances, and Welch tests were used. TrkC: F(5, 4.86) = 85.48, *p* = 9.61 × 10^−5^; Akt: F(5, 5.24) = 13.33, *p* = 5.49 × 10^−3^; PLC-γ: F(5, 5.36) = 12.06, *p* = 6.47 × 10^−3^; CREB: F(5, 5.46) = 30.19, *p* = 6.01 × 10^−4^. Post hoc pairwise comparisons were performed using Tamhane’s T2. TrkC: compared with the sham group, *p*_Model_ = 3.37 × 10^−2^; compared with the model group, *p*_Ketamine_ = 3.77 × 10^−3^, *p*_CAVO L_ = 4.09 × 10^−2^, *p*_CAVO M_ = 3.15 × 10^−1^, *p*_CAVO H_ = 3.74 × 10^−1^. Akt: compared with the sham group, *p*_Model_ = 2.35 × 10^−2^; compared with the model group, *p*_Ketamine_ = 7.56 × 10^−2^, *p*_CAVO L_ = 5.02 × 10^−1^, *p*_CAVO M_ = 2.78 × 10^−1^, *p*_CAVO H_ = 4.25 × 10^−1^. PLC-γ: compared with the sham group, *p*_Model_ = 6.79 × 10^−2^; compared with the model group, *p*_Ketamine_ = 1.78 × 10^−1^, *p*_CAVO L_ = 8.44 × 10^−1^, *p*_CAVO M_ = 6.18 × 10^−1^, *p*_CAVO H_ = 7.05 × 10^−1^. CREB: compared with the sham group, *p*_Model_ = 3.76 × 10^−2^; compared with the model group, *p*_Ketamine_ = 1.08 × 10^−1^, *p*_CAVO L_ = 5.64 × 10^−1^, *p*_CAVO M_ = 2.01 × 10^−1^, *p*_CAVO H_ = 3.71 × 10^−1^. The data of P75NTR, p38, and ERK1/2 were not normally distributed, and Kruskal–Wallis tests with post hoc analysis were used. P75NTR: H = 15.18, *p* = 9.64 × 10^−3^; p38: H = 16.16, *p* = 6.41 × 10^−3^; ERK1/2: H = 16.41, *p* = 5.77 × 10^−3^. Post hoc verification of P75NTR: compared with the sham group, *p*_Model_ = 1.32 × 10^−3^; compared with the model group, *p*_Ketamine_ = 2.86 × 10^−3^, *p*_CAVO L_ = 4.00 × 10^−1^, *p*_CAVO M_ = 7.86 × 10^−2^, *p*_CAVO H_ = 1.26 × 10^−1^. Post hoc verification of p38: compared with the sham group, *p*_Model_ = 5.79 × 10^−4^; compared with the model group, *p*_Ketamine_ = 5.91 × 10^−3^, *p*_CAVO L_ = 4.91 × 10^−1^, *p*_CAVO M_ = 6.65 × 10^−2^, *p*_CAVO H_ = 1.08 × 10^−1^. Post hoc verification of ERK1/2: compared with the sham group, *p*_Model_ = 7.61 × 10^−4^; compared with the model group, *p*_Ketamine_ = 7.41 × 10^−3^, *p*_CAVO L_ = 5.92 × 10^−1^, *p*_CAVO M_ = 4.67 × 10^−2^, *p*_CAVO H_ = 1.93 × 10^−1^.

To validate the transcriptomic changes in key signaling pathways identified by omics analyses, qPCR was performed to measure mRNA expression levels of neurotrophic factors and downstream signaling molecules in the prefrontal cortex. The mRNA expression levels of neurotrophin receptors (TrkA, TrkB, TrkC, p75NTR) in the prefrontal cortex are presented in Figure 7A. In comparison to the sham group, the model group demonstrated a considerable downregulation of TrkA, TrkB, and TrkC mRNA (*p* < 0.01) and upregulation of p75NTR mRNA (*p* < 0.01). Ketamine treatment elevated TrkA, TrkB, and TrkC expression and decreased p75NTR expression relative to the model group (*p* < 0.01). Both CAVO M and CAVO H groups exhibited increases in TrkA and TrkB mRNA compared to the model group (*p* < 0.05).

Figure 7B depicts the mRNA expression of signaling molecules p38, ERK1/2, NF-κB, and c-Jun. The model group showed decreased p38 and ERK1/2 mRNA levels and increased NF-κB and c-Jun mRNA relative to the sham group (*p* < 0.01). Ketamine treatment reversed these alterations, increasing p38 and ERK1/2 and reducing NF-κB and c-Jun (*p* < 0.05). The CAVO L group demonstrated no significant effect on p38 or ERK1/2 (*p* > 0.05), decreased NF-κB expression (*p* < 0.01), and no change in c-Jun levels (*p* > 0.05). The CAVO M group significantly decreased NF-κB and c-Jun (*p* < 0.01). The CAVO H group showed no effect on p38 (*p* > 0.05), increased ERK1/2 (*p* < 0.05), and decreased both NF-κB and c-Jun (*p* < 0.01).

Figure 7C demonstrates that the mRNA expression levels of Akt were decreased in the model group relative to the sham group (*p* < 0.05), showing no effect on PLC-γ and CREB (*p* > 0.05). The expression levels of Akt, PLC-γ, and CREB mRNA showed no statistical significance in the ketamine group and the model group (*p* > 0.05). The CAVO groups also showed no effect on Akt, PLC-γ and CREB mRNA expression compared to the model group (*p* > 0.05).

### 2.9. Western Blotting Results of CAVO on Prefrontal Cortex and Hippocampal Tissues of CUMS Rats

**Figure 8:**
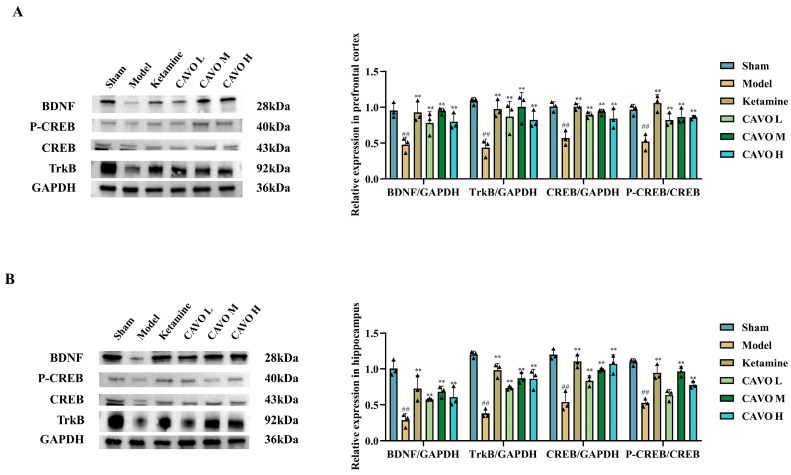
WB results of CAVO on the prefrontal cortex and hippocampus of CUMS rats. (**A**) BDNF, TrkB, CREB, and P-CREB protein expression in the prefrontal cortex of CUMS rats. Each group had 3 animals. Data that conformed to a normal distribution were presented as mean ± SD. Compared with the sham group, ^##^ *p* < 0.01. Compared with the model group, ** *p* < 0.01. The data of BDNF/GAPDH, TrkB/GAPDH, CREB/GAPDH and P-CREB/CREB were normally distributed and had homogeneous variances. Therefore, one-way ANOVA was used. BDNF/GAPDH: F(5, 12) = 7.17, *p* = 2.54 × 10^−3^; TrkB/GAPDH: F(5, 12) = 7.12, *p* = 2.61 × 10^−3^; CREB/GAPDH: F(5, 12) = 12.37, *p* = 2.15 × 10^−4^; P-CREB/CREB: F(5, 12) = 11.26, *p* = 3.37 × 10^−4^. Post hoc pairwise comparisons were performed using the LSD method. BDNF/GAPDH: compared with the sham group, *p*_Model_ = 3.23 × 10^−4^; compared with the model group, *p*_Ketamine_ = 5.17 × 10^−4^, *p*_CAVO L_ = 8.28 × 10^−3^, *p*_CAVO M_ = 3.75 × 10^−4^, *p*_CAVO H_ = 6.05 × 10^−3^. TrkB/GAPDH: compared with the sham group, *p*_Model_ = 1.85 × 10^−4^; compared with the model group, *p*_Ketamine_ = 8.56 × 10^−4^, *p*_CAVO L_ = 4.02 × 10^−3^, *p*_CAVO M_ = 5.55 × 10^−4^, *p*_CAVO H_ = 8.25 × 10^−3^. CREB/GAPDH: compared with the sham group, *p*_Model_ = 2.19 × 10^−5^; compared with the model group, *p*_Ketamine_ = 2.37 × 10^−5^, *p*_CAVO L_ = 3.66 × 10^−4^, *p*_CAVO M_ = 1.27 × 10^−4^, *p*_CAVO H_ = 1.34 × 10^−3^. P-CREB/CREB: compared with the sham group, *p*_Model_ = 8.62 × 10^−5^; compared with the model group, *p*_Ketamine_ = 1.44 × 10^−5^, *p*_CAVO L_ = 2.10 × 10^−3^, *p*_CAVO M_ = 7.68 × 10^−4^, *p*_CAVO H_ = 8.67 × 10^−4^. (**B**) BDNF, TrkB, CREB, and P-CREB protein expression in the hippocampus of CUMS rats. Each group had 3 animals. Data that conformed to a normal distribution were presented as mean ± SD. Compared with the sham group, ^##^ *p* < 0.01. Compared with the model group, ** *p* < 0.01. The data of BDNF/GAPDH, TrkB/GAPDH, CREB/GAPDH and P-CREB/CREB were normally distributed and had homogeneous variances. Therefore, one-way ANOVA was used. BDNF/GAPDH: F(5, 12) = 12.87, *p* = 1.78 × 10^−4^; TrkB/GAPDH: F(5, 12) = 34.65, *p* = 9.95 × 10^−7^; CREB/GAPDH: F (5, 12) = 19.16, *p* = 2.40 × 10^−5^; P-CREB/CREB: F(5, 12) = 28.54, *p* = 2.88 × 10^−6^. Post hoc pairwise comparisons were performed using the LSD method. BDNF/GAPDH: compared with the sham group, *p*_Model_ = 4.83 × 10^−6^; compared with the model group, *p*_Ketamine_ = 4.53 × 10^−4^, *p*_CAVO L_ = 8.71 × 10^−3^, *p*_CAVO M_ = 1.03 × 10^−3^, *p*_CAVO H_ = 4.72 × 10^−3^. TrkB/GAPDH: compared with the sham group, *p*_Model_ = 3.13 × 10^−8^; compared with the model group, *p*_Ketamine_ = 9.10 × 10^−7^, *p*_CAVO L_ = 1.67 × 10^−4^, *p*_CAVO M_ = 7.61 × 10^−6^, *p*_CAVO H_ = 9.24 × 10^−6^. CREB/GAPDH: compared with the sham group, *p*_Model_ = 1.84 × 10^−6^; compared with the model group, *p*_Ketamine_ = 9.06 × 10^−6^, *p*_CAVO L_ = 2.52 × 10^−3^, *p*_CAVO M_ = 8.52 × 10^−5^, *p*_CAVO H_ = 1.67 × 10^−5^. P-CREB/CREB: compared with the sham group, *p*_Model_ = 3.87 × 10^−7^; compared with the model group, *p*_Ketamine_ = 9.22 × 10^−6^, *p*_CAVO L_ = 7.89 × 10^−2^, *p*_CAVO M_ = 5.95 × 10^−6^, *p*_CAVO H_ = 9.32 × 10^−4^.

To confirm the protein-level changes in the NT/TrkB signaling pathway, Western blotting was performed to detect the expression levels of BDNF, TrkB, CREB, and phosphorylated CREB in the prefrontal cortex and hippocampus. Protein expression levels of BDNF, TrkB, CREB, and p-CREB in the prefrontal cortex are presented in Figure 8A. All four proteins were markedly downregulated in the model group relative to the sham group (*p* < 0.01). Ketamine therapy markedly increased their expression compared to the model group (*p* < 0.01). The CAVO L group exhibited increased BDNF and TrkB expression (*p* < 0.05), with significant elevations in CREB and p-CREB (*p* < 0.01). The CAVO M group showed significant increases in all four proteins (*p* < 0.01). The CAVO H group demonstrated significant elevations in BDNF, TrkB, and CREB (*p* < 0.05), and p-CREB (*p* < 0.01).

Similar expression patterns were observed in hippocampal tissues (Figure 8B). Levels of BDNF, TrkB, CREB, and p-CREB were reduced in the model group relative to the sham group (*p* < 0.01). Ketamine treatment significantly increased their expression levels (*p* < 0.01). The CAVO L group exhibited increased BDNF, TrkB, and CREB expression (*p* < 0.05), with no significant change in p-CREB (*p* > 0.05). Both CAVO M and CAVO H groups significantly increased the expression of all four proteins relative to the model group (*p* < 0.01).

## 3. Discussion

In CUMS model rats, inhaling aerosolized CAVO significantly reduced brain pathology and depression-like behaviors. CAVO significantly changed serum levels of inflammatory cytokines and neurotrophic factors. Metabolomics identified 25 biomarkers associated with the antidepressant effects of CAVO, involving multiple metabolic pathways. Further analysis through transcriptomics and proteomics indicated that CAVO exerts its antidepressant effects via the NT-Trk signaling pathway. Functional analyses highlighted key molecular players such as BDNF, TrkB, and CREB, with qPCR and Western blot validation confirming their close association with the antidepressant action of CAVO. Thus, by modulating the NT/Trk signaling pathway, CAVO ameliorates depression-like behaviors in CUMS rats. These findings highlight the therapeutic potential of CAVO and establish a basis for further investigation and advancement of TCM-based treatments for depression.

According to recent research, there is a substantial overlap between olfactory processing and brain areas like the hippocampus that regulate emotions [14]. Olfactory sensory input can be projected to structures such as the prefrontal cortex and hippocampus, which play crucial roles in emotional regulation and stress response [15]. Functional imaging studies have demonstrated that olfactory stimuli can modulate the activity in these regions, influencing the autonomic nervous system output and emotional states [16]. A growing body of evidence suggests that the olfactory system may represent a promising therapeutic target for depression, with several studies reporting antidepressant-like effects following olfactory enrichment or stimulation [17,18]. Volatile oils derived from traditional natural herbs are commonly used to alleviate depression and stabilize mood, including lavender essential oil [19,20] and Acorus tatarinowii essential oil [21]. These volatile compounds can easily cross the blood–brain barrier, exerting effective antidepressant effects. Furthermore, owing to their reduced toxicity and diminished side effects, essential oils are broadly embraced, and their modes of administration are more convenient than those of traditional antidepressants. In summary, essential oils serve as a valuable adjuvant therapy in depression treatment.

Behavioral assessments indicated that rats exposed to CUMS display depressive-like symptoms, including diminished locomotor activity, lowered food preference, and heightened immobility in both the open-field test and forced swim test. Histopathological analyses reveal specific neuronal damage within the hippocampus. However, treatments with ketamine and CAVO significantly alleviated depressive behaviors and repaired hippocampal neuronal injuries. These findings suggest that CAVO has antidepressant properties. To clarify the underlying mechanisms, we conducted proteomics and metabolomics analyses on hippocampus samples from rats. The results indicate that CAVO may exert its effects through the neuroactive ligand receptor interaction pathway. Therefore, our focus was directed toward investigating the NT-Trk signaling pathway.

CAVO effectively reverses the core neurotrophic signaling deficiencies induced by CUMS. The prefrontal cortex and hippocampus are essential brain regions that regulate emotion and cognition, serving as crucial locations for BDNF/TrkB signaling expression [22,23]. Our findings reveal that, in the CUMS rat model, there is downregulation of BDNF and its high-affinity receptor TrkB protein expression in these areas, accompanied by reductions in the downstream effectors CREB and its phosphorylated form p-CREB. This phenomenon aligns with the classical theory of weakened neurotrophic support in depression [24]. Notably, CAVO treatment, especially at medium and high doses, significantly elevates the levels of these proteins. This suggests that CAVO directly enhances the initiation and termination stages of NT-Trk signaling pathways, providing a molecular basis for the subsequent promotion of neuronal survival and plasticity. Furthermore, CAVO exerts bidirectional and finely tuned transcriptional regulation of the NT-Trk pathway. Quantitative PCR results further demonstrate the systemic effects of CAVO; it not only upregulates mRNA expression of Trk family receptors TrkA/B/C and downstream molecules Akt, ERK1/2, CREB, and PLC-γ but also markedly suppresses the transcription of stress- and inflammation-related genes P75NTR, NF-κB, and c-Jun. As a low-affinity receptor for neurotrophic factors, P75NTR is often overactivated under chronic stress, shifting signaling toward c-Jun and NF-κB pathways that promote apoptosis and neuroinflammation [25,26]. Therefore, CAVO plays a dual role of enhancing regulation in classical Trk-mediated pro-survival signals while suppressing P75NTR-mediated pro-apoptotic and inflammatory pathways. This function is pivotal in correcting the neural signaling network imbalance caused by CUMS.

The central regulatory pathways of CAVO are corroborated by its systemic peripheral effects. Our study indicates that CAVO not only improves central nervous system molecular expression but also reverses elevated serum pro-inflammatory cytokines IL-1, IL-6, and TNF-α induced by CUMS. It concurrently raises levels of monoaminergic neurotransmitters 5-HT and DA, along with neurotrophic factors BDNF, NT-3, and NT-4. Activation of the NT-Trk pathways centrally, particularly through MAPK/ERK and PI3K/Akt signaling, may inhibit microglia-mediated neuroinflammation, potentially accounting for the observed reduction in peripheral inflammation [27,28]. Additionally, neurotrophic signals closely interact with the monoaminergic system; BDNF-TrkB signaling promotes the function and survival of serotonergic and dopaminergic neurons [29,30]. Although serum neurotrophins and cytokine levels served as accessible peripheral biomarkers that aligned with central findings, the primary mechanistic conclusions of this study are based on brain tissue analyses. Consequently, the systemic improvements reflected in peripheral blood markers are likely secondary to modulation of the central NT-Trk pathway by CAVO, which forms the foundation of its antidepressant phenotype.

Moreover, we observed that the antidepressant effects of CAVO are dose-dependent. Medium and high concentrations of CAVO significantly ameliorated multiple depression-related metrics, including neuroprotection, serum neurotransmitter levels, and NT-Trk pathway expression, indicating enhanced therapeutic efficacy with increasing dose within an optimal concentration range.

## 4. Materials and Methods

### 4.1. Preparation of CAVO

Steam distillation was employed to extract the raw materials provided by Yunnan Hehe Chinese Herbal Medicine Slices Co., Ltd. (Kunming, China). The herbs were proportionately weighed, pounded into a powder, mixed, and immersed in water at eight times their weight for four hours. The total yield of essential oils was 3.6% (*v*/*w*). The extract was desiccated with anhydrous sodium sulfate and stored at 4 °C in amber glass vials [31].

### 4.2. Establishment of CUMS Rat Model and Drug Treatment

Sprague–Dawley rats, male, 180 ± 20 g, were acquired from SPF (Beijing) Biotechnology Co., Ltd. (SCXK, Beijing, China, 2024-0001). The experiment was located in the animal room of Yunnan University of Chinese Medicine. The rats were kept in cages with closely regulated experimental conditions, including a one-week acclimatization period, a 12 h light/12 h dark cycle with lighting from 7:00 AM to 7:00 PM, a temperature of 25 ± 1 °C, daily bedding changes, and with free ad libitum access to food and drinking water. The Yunnan University of Chinese Medicine Medical Ethics Committee approved all rat procedures (R-062023221).

The rats in the experimental group were housed individually in standard cages to prevent social interaction, whereas the rodents in the sham experimental group were co-housed. As previously mentioned, the following nine stressors were introduced at random during the 35-day model’s setup: (1) reversal of light/dark cycle for 24 h; (2) cage inclination (without bedding) for 12 h; (3) tail nip (0.5–1 cm from the tail tip) for 1 min; (4) hot water immersion (45 °C) for 5 min; (5) cold water immersion (5 °C) for 5 min; (6) moist bedding (300 mL of water per cage) for 24 h; (7) deprivation of food and drink for 24 h; (8) shaking the rat cage at a frequency of once per second for 1 min; and (9) placement of foreign objects in the rat cage for 24 h. To prevent stress habituation, irregular pressure stimulation was employed throughout the establishment of the CUMS model [32,33].

**Figure 9:**
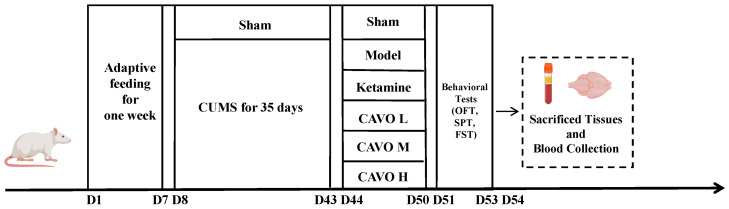
Schematic diagram of modeling and drug intervention time flow.

As stated below, all rats were divided into six groups using a random number table method (*n* = 10): sham group; CUMS model group, administered physiological saline at a dosage of 8 mL/kg; positive drug control group, receiving ketamine at 10 mg/kg; high-dose CAVO group, designated as CAVO H, at 40 mg/kg; medium-dose CAVO group, referred to as CAVO M, at 20 mg/kg; and low-dose CAVO group, identified as CAVO L, at 10 mg/kg. The above drugs (including physiological saline, ketamine, and CAVO) were administered continuously for 1 week, which were administered via nebulized inhalation [34]. All animals were administered the drugs at the same time each day. The timeline for model establishment and drug treatment is shown in Figure 9. And the sample size for each endpoint of the experiment is shown in Table 2.

### 4.3. Behavior Tests

#### 4.3.1. Sucrose Consumption Test

During the CUMS period, tests were administered once a week using the previously described methodology. To acclimate the rats to the sucrose solution (1%, *w*/*v*), two bottles—one containing tap water and the other containing a 1% sucrose solution—were positioned in each cage for a whole day. The rats were deprived of food and water for 24 h before being allowed free access to the bottles following their acclimation. The sucrose preference rate was calculated using the formula: sucrose solution (g)/(sucrose solution [g] + water [g]) × 100%.

#### 4.3.2. Open-Field Test (OFT)

The experimental apparatus, supplied by Shanghai Xinsuan Information Technology Co., Ltd. (Shanghai, China). The open-field reaction chamber has a square base that is 100 cm on each side and is about 40 cm tall. There are 25 little squares on the base, each measuring 4 cm on each side, and the inside walls are painted black. A digital camera is mounted 2 m above the top of the chamber to capture a complete view of the open field’s internal space. External interference is minimized, with laboratory background noise controlled below 65 decibels. During the experiment, the rat is gently placed at the center of the bottom surface of the reaction box, and the camera and timing programs are simultaneously activated. After continuous observation for 5 min, the recording is stopped. After each test, to prevent residual urine, feces, or odors from the previous experiment from interfering with subsequent results, the reaction box’s bottom surface and interior walls were completely cleaned. After cleaning, a new experimental rat is placed, and the experiment repeated, with data collected and analyzed by the automatic data collection and processing system.

#### 4.3.3. Forced Swim Test (FST)

The experimental apparatus consisted of a cylindrical barrel of 60 cm in height and 28 cm in width, filled with 30 cm of water kept at a temperature of 25 ± 1 °C. Prior to the formal experiment, each rat was gently placed into the barrel, ensuring that its limbs and tail did not touch the bottom, and allowed to swim adaptively for 15 min in a quiet state. Immediately following adaptation training, the rats were taken out of the barrel, gently patted dry with a towel, and put back in their original cages. During the formal testing phase, the rats were again placed in the aforementioned barrel, and each rat was observed for 6 min. A camera was used to precisely record the cumulative immobility time of the rats over the final 5 min. After the test, the rats were dried in the same manner and returned to their cages. The forced swimming test effectively assesses adaptive capacity to adverse environments. Through behavioral performance, it directly reflects despair behavior and depressive states, providing critical data support for studying related physiological and psychological mechanisms.

### 4.4. Tissue Sample Collection and Histopathology Experiments

After blood was collected, rats were sacrificed via cervical dislocation. The hippocampus and prefrontal cortex were promptly excised, placed on ice, and subsequently stored at −80 °C. Additionally, three rats were taken out of each group, and the brain tissue was preserved for 48 h in formalin. A microtome (RM2245, Shanghai Leica Instrument Co., Ltd., Shanghai, China) was used to gather and segment the hippocampal tissue (4 μm). Lastly, Nissl staining and hematoxylin and eosin (HE) staining were carried out. Neutral resin was then used to mount the stained portions onto a slide. Using a 100× optical microscope (BX43F, Olympus Corporation, Tokyo, Japan), the hippocampal nerve damage in the CA1 areas of each rat was observed and captured on camera.

Because the hippocampal samples were fully used for histopathology, metabolomics, and proteomics (with metabolomics and proteomics sharing the same five animals), no hippocampal tissue remained for RT-qPCR. Therefore, the prefrontal cortex, a brain region also critically involved in emotional regulation and depression pathophysiology, was collected from separate animal subgroups for RT-qPCR and Western blot validation (as indicated in Table 2).

### 4.5. Multi-Omics Analysis

#### 4.5.1. Metabolomics

Samples were thawed at 4 °C for metabolite extraction, and then 400 μL of a 4:1 methanol:water solution that had been pre-cooled was added; each sample was weighed at 50 mg, and two steel beads were added. After being broken down and homogenized at low temperature in a tissue grinder, the samples were combined with a methanol–water solution, sonicated for 20 min in an ice bath, and then stood at −20 °C for one hour. The samples were subsequently centrifuged for 20 min at 4 °C at 16,000× *g*, and the supernatant was collected.

The sample was positioned in liquid chromatography–mass spectrometry (LC-MS/MS) (TSQ Quantis Plus, Thermo Fisher Scientific, Waltham, MA, USA), with the injection volume calibrated to 4 μL to ensure precision and consistency in the injection volume. The flow rate was controlled at 0.3 mL/min to ensure sufficient separation time for the sample within the column while avoiding separation efficiency issues caused by excessive or insufficient flow rates. In order to establish an appropriate temperature environment for metabolite separation in the chromatographic column, the column temperature was kept at 40 °C. A gradient elution program was employed for efficient metabolite separation as follows: from 0–2 min, the B mobile phase ratio was 0%; 2–6 min, linear increases from 0% to 48%; 6–10 min, linear increases from 48% to 100%; 10–12 min, B solution ratio maintained at 100%; 12–12.1 min, linear decrease from 100% to 0%; 12.1–15 min, maintained at 0%. We completed each full elution cycle and separated the metabolites of each property one by one. Regarding ionization mode and instrument parameters, each sample was separated by liquid chromatography and analyzed in both positive ion (+) and negative ion (−) modes to comprehensively cover metabolites of different polarities. A QE Plus mass spectrometer with a HESI source for ionization was utilized for the mass spectrometry analysis. Ionization parameters were set at 3.8 kV for positive ion mode and 3.2 kV for negative ion mode. The mass spectrometry acquisition time was set to 15 min to ensure complete collection of mass spectrometry signals for all metabolites in the sample. The parent ion scan range was set to 75–1050 *m*/*z*, comprehensively covering the possible mass range of metabolite ions, providing robust support for structural identification and quantitative analysis of metabolites. MS-DIAL for peak detection; QC samples with CV > 30% excluded; PCA and OPLS-DA using SIMCA-P 14.1; differential metabolites filter based on VIP > 1 and FDR-corrected *p* < 0.05; KEGG enrichment using MetaboAnalyst 5.0 (hypergeometric test, FDR < 0.05).

#### 4.5.2. Proteomics

For protein extraction, we placed the sample in liquid nitrogen and ground it thoroughly to break down the tissue cells. After properly mixing each sample with the required amount of SDT lysis solution, the samples were moved to an Eppendorf (EP) tube. The proteins were then denatured and made more soluble by immersing the EP tube in a hot water bath for three minutes. A 2 min ultrasonic disruption was immediately performed to further disrupt the cellular structure and release more proteins. Thereafter, the EP tube was placed in a 4 °C environment and centrifuged at 16,000× *g* for 20 min. The protein concentration in the supernatant was precisely measured by aspirating it carefully and using the bicinchoninic acid (BCA) method, providing a protein sample with uniform concentration for subsequent experiments.

Following protein quantification, a suitable number of peptide segments were accurately quantified for each sample. Using the Neo UHPLC system controlled by the Vanquish Neo UHPLC system, chromatographic separation of the peptide segments was performed. Gradient separation was carried out according to the pre-set liquid phase gradient program. The specific settings of this gradient program were as follows: within 0–0.1 min, the proportion of B solution was linearly increased from 4% to 6%; 0.1–1.1 min, increased from 6% to 12%; 1.1–4.3 min, increased from 12% to 22.5%; 4.3–6.1 min, linear increments from 22.5% to 45%; 6.1–8 min, maintained at 99%. Mass spectrometry analysis was performed on the peptide segments that had been isolated by chromatography. To complete the protein DIA quantitative analysis and DIA mass spectrometry data database retrieval, all mass spectrometry data were combined using DIA-NN software. The database is https://www.uniprot.org/taxonomy/10116 (accessed on 3 May 2024). DIA-NN (v1.8) against UniProt database; MaxLFQ normalization; one-way ANOVA (*p* < 0.05) for differential expression; GO and KEGG enrichment using clusterProfiler (hypergeometric test, BH-adjusted *p* < 0.05).

### 4.6. Enzyme-Linked Immunosorbent Assay (ELISA)

The blood samples were stored at −80 °C for later use after being centrifuged for 15 min at 3500 rpm to obtain serum. According to the manufacturer’s instructions, commercial ELISA kits (Jiangsu adsbio, Yancheng, China) were used to measure the levels of stress hormones (cortisol), inflammatory factors, neurotransmitters, and neurotrophic factors in the serum. The optical density of each well was measured with an ELISA reader calibrated to 450 nm.

### 4.7. Reverse Transcription–Quantitative Polymerase Chain Reaction (RT-qPCR)

Prefrontal cortex tissue was removed and homogenized in a tube until no visible tissue chunks remained. We extracted total RNA using Trizol. Following the instructions of the reverse transcription kit, the reverse transcription reaction system was prepared to obtain the cDNA, which was used as a template for RT-PCR amplification. The formulation of the retrotranscriptional reaction system was as follows: 4 μL of 5 × Reaction Buffer, 0.5 μL of Oligo (dT)18 Primer, 0.5 μL of Random Hexamer primer, 1 μL of Servicebio^®^RT Enzyme Mix, 10 μL of total RNA, and RNase-free water was added to a total volume of 20 μL. The reverse transcription conditions were set as follows: 25 °C for 5 min, 42 °C for 30 min, and 85 °C for 5 s. For PCR amplification, a 0.2 mL PCR tube was used to prepare a 7.5 μL of Mix (None ROX), 1.5 μL of gene primer (upstream + downstream), 2 μL of the reverse transcription product (cDNA), and 4 μL of ddH2O. The RT-PCR amplification conditions were as follows: 95 °C for 30 s for predenaturation, followed by 95 °C for 15 s for denaturation, 60 °C for 30 s for annealing/extension, and a temperature range from 65 °C to 95 °C with each temperature rise of 0.5 °C, collecting a fluorescence signal at each step. After amplification, the 2^−ΔΔCT^ value was calculated [35]. The primer names and sequences can be found in Table 3.

### 4.8. Western Blotting

Total protein was extracted with RIPA lysis buffer and measured utilizing the Immobilon BCA assay kit. Proteins were extracted via 12% Sodium Dodecyl Sulfate-Polyacrylamide Gel Electrophoresis and subsequently transferred to a Polyvinylidene Difluoride membrane (Immobilon). The primary antibody (1:1000, Proteintech, Wuhan, China) was incubated at 4 °C for the entire night following an hour of membrane blocking at 25 °C with 5% non-fat milk. The secondary antibody (1:10,000, Proteintech, Wuhan, China) was applied to the membrane and incubated for an additional hour. The iBright FL 1500 (Thermo Fisher Scientific, Waltham, MA, USA) was used for detection. The relative expression levels of the associated proteins were computed by measuring and analyzing the bands’ gray values using ImageJ 1.54f image analysis software.

### 4.9. Statistical Analysis

The data were analyzed using IBM SPSS Statistics 31.0.2.0 and graphs were drawn using GraphPad Prism 9.0 software. Shapiro–Wilk tests were used for verifying the normality assumption. *p* > 0.05 was considered a normal distribution. If the data followed a normal distribution, Levene tests were used for the homogeneity of variance. If the variances were equal, one-way ANOVA tests were used and post hoc multiple comparisons were performed using LSD. If the variances were not uniform, the Welch test was used and multiple comparisons were performed using Tamhane’s T2. If the data did not follow a normal distribution, Kruskal–Wallis tests with post hoc analysis were used. *p* < 0.05 was considered to indicate a statistically significant result. Data that conformed to a normal distribution were presented as mean ± standard deviation (SD). Data that did not follow a normal distribution were expressed as median and interquartile range (IQR).

## 5. Limitations

Several limitations of this study should be acknowledged. First, although the chemical composition of CAVO has been described [13], the specific active components responsible for the antidepressant effects remain to be determined. Essential oils are complex mixtures in which multiple constituents often act synergistically rather than through a single molecule. Future studies will employ network pharmacology combined with bioactivity-guided fractionation to identify the key active components and elucidate their individual contributions. Second, metabolomics and proteomics were performed on the hippocampus, while RT-qPCR was performed on the prefrontal cortex due to tissue yield constraints. Subsequent work should validate the omics findings in the prefrontal cortex using targeted approaches. Third, serum levels of neurotransmitters and neurotrophins were measured as complementary peripheral biomarkers, while the primary mechanistic evidence (BDNF, TrkB, CREB) was obtained from brain tissue. Future studies should directly measure monoamine neurotransmitters in brain tissue to further validate central-peripheral correlations. Fourth, current research lacks in vitro mechanistic validation. Future studies should involve constructing gene-edited cell models to explore the relationship between CAVO and NT-Trk signaling pathways at the cellular level.

## 6. Conclusions

CAVO alleviates depressive-like behaviors and hippocampal neuronal damage in CUMS rats, reduces peripheral neuroinflammatory cytokines, modulates monoamine neurotransmitters, and improves neurotrophic factor expression. The NT-Trk signaling pathway appears to be the primary mechanistic basis for these antidepressant effects, functioning through the augmentation of BDNF/TrkB and downstream Akt/ERK/CREB axes to promote neuroplasticity and cell survival, while concurrently suppressing P75NTR/NF-κB-mediated stress, constructing gene-edited cell models’ responses to mitigate neuroinflammation. This comprehensive pharmacological insight lays a robust foundation for the development of CAVO as a potential antidepressant agent.

## Figures and Tables

**Table 1:** Metabolite information of CAVO-intervened CUMS rats.

No.	Alignment ID	Metabolite Name	Rt (min)	Experiment Mz	Reference *m*/*z*
1	NEG4455	N-Acetylneuraminic acid	0.902	308.098	308.099
2	POS524	Choline	10.362	104.108	104.107
3	NEG13003	PE(P-18:0/18:1)	11.366	728.558	728.560
4	POS8501	Calicoferol G	11.705	399.326	399.326
5	NEG4413	Glutathione	4.874	306.076	306.076
6	POS1634	Gallic acid	6.869	153.019	153.018
7	NEG10743	UDP-glucose	0.975	565.047	565.048
8	POS13592	Oboflavanone B	11.001	553.257	553.256
9	POS8099	Persicaxanthin	9.310	385.274	385.274
10	POS798	1-(Diethylamino)ethanol	1.330	118.123	118.123
11	POS824	Indane	8.937	119.086	119.086
12	NEG3983	SedohePtulose 7-PhosPhate	0.778	289.032	289.032
13	POS5388	Phenyl 2-acetamido-2-deoxy-alPha-D-glucoPyranoside	4.920	298.130	298.129
14	POS4426	Graveolide	7.777	266.174	266.175
15	NEG677	Aspartate	0.859	132.028	132.030
16	POS2662	Cycluron	8.070	199.181	199.180
17	POS5731	Glutathione (Reduced)	1.456	308.092	308.091
18	NEG1015	L-Histidine	0.827	154.060	154.062
19	NEG6712	N-Stearoyl Taurine	10.981	390.267	390.268
20	NEG432	4-Ethynylaniline	5.214	116.049	116.051
21	POS2439	Targinine	0.883	189.135	189.135
22	NEG1947	3h-Indole-3-ProPanoic acid, a-amino	5.216	203.081	203.083
23	NEG3472	3-Oxohexadecanoic acid	11.043	269.211	269.212
24	POS5928	CHEBI:79180	9.707	313.274	313.274
25	POS20174	PC (18:2/18:2)	8.843	782.569	782.569

**Table 2:** Sample size allocation for each experimental endpoint.

Experimental Endpoint	Tissues/Samples Used	Sample Size (per Group)	Specific Animal IDs (Within Group)
Behavioral tests (SPT, OFT, FST)	Whole animals	10	#1–10
ELISA	Blood serum	10	#1–10
Histopathology (HE and Nissl staining)	Hippocampus	3	#1, #2, #3
Metabolomics and proteomics	Hippocampus	5	#4, #5, #6, #7, #8
RT-qPCR	Prefrontal cortex	3	#8, #9, #10
Western blot	Prefrontal cortex and hippocampus	3	#8, #9, #10

#: the label of each group of rats.

**Table 3:** Primer information.

Name	Sequence (5′–3′)	Product Length (bp)
Ntrk1-F	AGGAGGATTTGTGTGGTGTGTAT	127
Ntrk1-R	GAGTCATTGGGCATCTGGATCTT	127
Ntrk2-F	CGGATGACAGTGGGAAACAAATC	195
Ntrk2-R	CTCCGTTGTAGAACCACTGAAGT	195
Ntrk3-F	CAAGCCCACCCACTACAACA	152
Ntrk3-R	GTGTAGGGCTCGCATCAGAC	152
Ngfr-F	TGCCTGGACAGTGTTACATTCTC	160
Ngfr-R	CAGTCTCCTCGTCCTGGTAGTA	160
Akt-F	CCTTCCTTACAGCCCTCAAGTAC	184
Akt-R	TTCTTCTCGGAGTGCAAGTAGTC	184
Nfkb1-F	GCGTCCAACCTGAAGATCGTAAG	151
Nfkb1-R	ATCCTTCCCAAACTCCACCATTT	151
Plcg1-F	GGCTTCAGGTGCAGGAATTTATG	171
Plcg1-R	ACAGTGGGTTGTTCATGGTTTCT	171
Creb-F	TTCAAGCTGCCTCTGGTGATGTA	167
Creb-R	TGCTGCTTCCCTGTTCTTCATTA	167
Jun-F	CGCACGCTCCTAAACAAACTTTG	156
Jun-R	GTCGTTTCCATCTTTGCAGTCAT	156
Mapk14-F	CACCAACCATTGAGCAGATGAAA	176
Mapk14-R	GAGGTCACGGTGCAGAACATTAG	176
Mapk3/Mapk1-F	TTGACATGGAGCTGGATGATCTC	192
Mapk3/Mapk1-R	TCCGGGTTGAGCAAAGTTCATTT	192
GAPDH-F:	GAAGGTCGGTGTGAACGGAT	251
GAPDH-R:	CCCATTTGATGTTAGCGGGAT	251

Gene symbols follow the official nomenclature of the NCBI Gene database for Rattus norvegicus. Commonly used aliases are provided in parentheses for clarity: Ntrk1 (TrkA), Ntrk2 (TrkB), Ntrk3 (TrkC), Ngfr (p75NTR), Nfkb1 (NF-κB), Plcg1 (PLC-γ), Creb (CREB), Jun (c-Jun), Mapk14 (p38), Mapk3 (ERK1)/Mapk1 (ERK2).

## Data Availability

The original contributions presented in this study are included in the article. Further inquiries can be directed to the corresponding author.
